# Supplementary material for: Altered microRNA Transcriptome in Cultured Human Liver Cells upon Infection with Ebola Virus
Source: Int J Mol Sci. 2021 Apr 6;22(7):3792. doi: 10.3390/ijms22073792 (PMC8038836; doi:10.3390/ijms22073792)
Supplement: Supplementary file 1 [file ijms-22-03792-s001.zip › Supplementary_File/C_ GO_Analysis_Results/16-30nt_go_Reston-96h-Huh7_vs_Control-96h-Huh7_up.mature_mirna_targets/BP_FoldEnrichment.pdf]

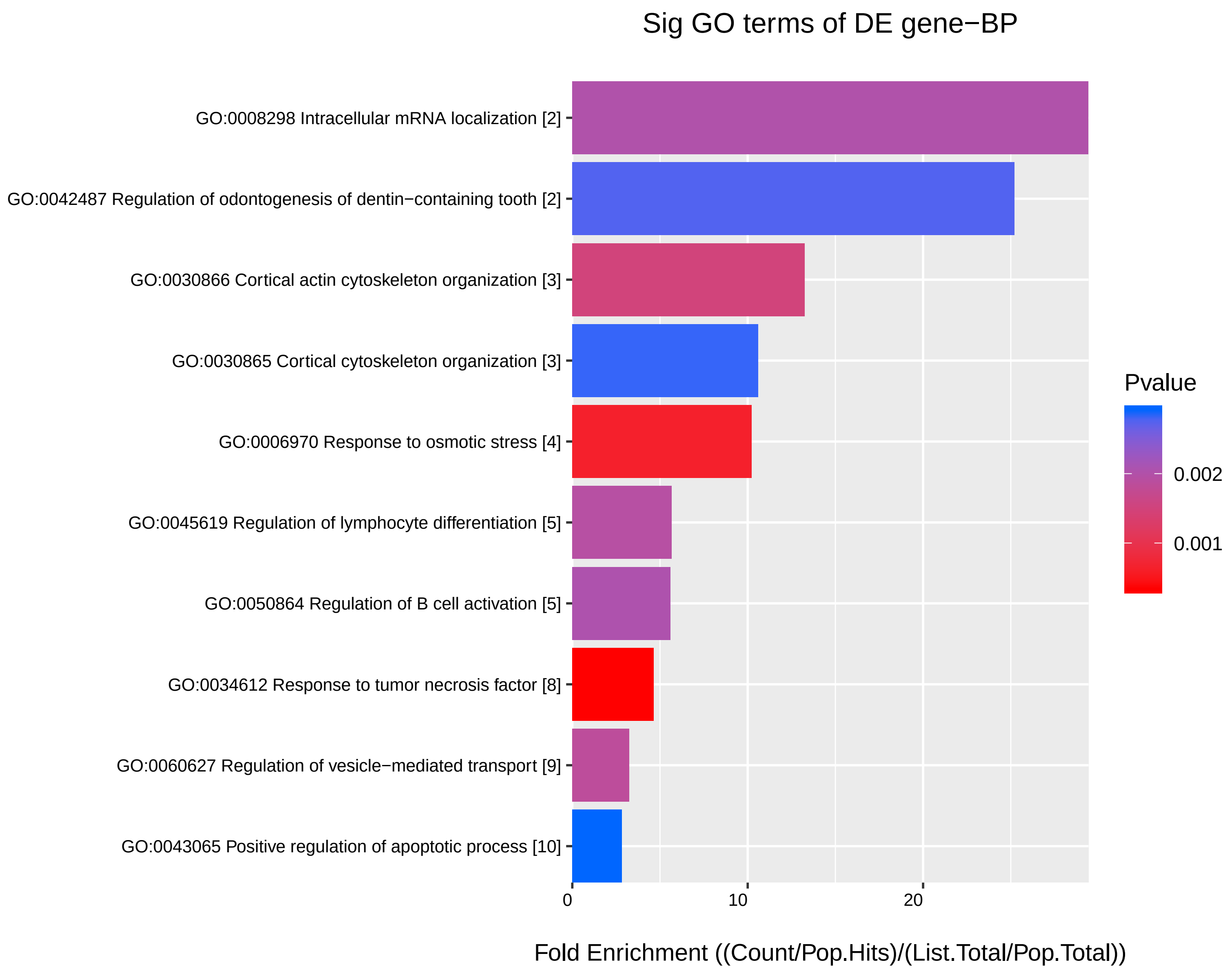
Sig GO terms of DE gene−BP
GO:0008298 Intracellular mRNA localization [2]
GO:0042487 Regulation of odontogenesis of dentin−containing tooth [2]
GO:0030866 Cortical actin cytoskeleton organization [3]
GO:0030865 Cortical cytoskeleton organization [3]
GO:0006970 Response to osmotic stress [4]
GO:0045619 Regulation of lymphocyte differentiation [5]
GO:0050864 Regulation of B cell activation [5]
GO:0034612 Response to tumor necrosis factor [8]
GO:0060627 Regulation of vesicle−mediated transport [9]
GO:0043065 Positive regulation of apoptotic process [10]
0
10
20
Fold Enrichment ((Count/Pop.Hits)/(List.Total/Pop.Total))
Pvalue
0.002
0.001
